# Phytotherapy in precision medicine era: deciphering herbal modulation of multi-omic biomarkers for cardiovascular metabolic syndrome remodeling

**DOI:** 10.3389/fmed.2026.1790060

**Published:** 2026-05-20

**Authors:** Xiao-hong Yu, Qi-yue Zhao, Peng Fu, Yi-he Tang, Wen-wei Wang, Xi-wen Yu, Ya-xiong Sun

**Affiliations:** 1 Second Department of Cardiology, First Affiliated Hospital of Heilongjiang University of Chinese Medicine, Harbin, China; 2 The First School of Clinical Medicine of Heilongjiang University of Chinese Medicine, Harbin, China; 3 Department of Preventive Treatment, First Affiliated Hospital of Heilongjiang University of Chinese Medicine, Harbin, China; 4 Department of Rehabilitation, First Affiliated Hospital of Heilongjiang University of Chinese Medicine, Harbin, China; 5 Department of Acupuncture and Moxibustion, Baicheng Medical College, Baicheng, China; 6 Department of Cardiology, Hohhot Hospital of Traditional Chinese Medicine and Mongolian Medicine, Hohhot, China

**Keywords:** biomarkers, cardiometabolic syndrome, cardiovascular remodeling, multi-omics, phytotherapy, precision medicine

## Abstract

This perspective article examines the integration of traditional phytotherapy into precision medicine for cardiovascular remodeling associated with cardiometabolic syndrome (CMS). Herbal medicine, with its intrinsic multi-target action, aligns well with the complex network pathology of CMS. However, challenges remain in standardizing its use and elucidating its mechanisms. We argue that multi-omics biomarkers—spanning genomics, transcriptomics, proteomics, metabolomics, and microbiomics—are essential for systematically deciphering the systemic effects of herbal interventions. By identifying specific biomarker profiles that predict efficacy, stratify patients, and monitor treatment response, traditional herbal therapy can evolve from a holistic practice into an evidence-based, personalized approach. This strategy offers a rigorous, scientifically grounded pathway to individualized cardiovascular remodeling management, effectively bridging empirical wisdom with contemporary precision medicine.

## 1 Introduction

### 1.1 Complex network of cardiometabolic remodeling

Cardiometabolic syndrome (CMS) is a cluster of interrelated metabolic risk factors, including central obesity, insulin resistance, hypertension, dyslipidemia, and hyperglycemia (1, 2). These components collectively drive cardiovascular structural and functional remodeling through intertwined mechanisms such as chronic low-grade inflammation, metabolic dysregulation, oxidative stress, and fibrosis, ultimately leading to heart failure and atherosclerosis (3–5). This syndrome represents a highly heterogeneous and dynamic pathological network, with significant variation in clinical presentation among individuals (2). Conventional single-target therapies, while effective, often address only one aspect of this complex network and may be insufficient to halt systemic remodeling driven by multi-system dysfunction. Consequently, there is a pressing need for multi-targeted, personalized intervention strategies that can modulate the underlying network pathology (1, 2).

### 1.2 Phytotherapy: bridging tradition and precision medicine

Phytotherapy has a well-documented historical and empirical foundation in cardiovascular health management. Its contemporary relevance lies in its inherent “multi-component, multi-target, multi-pathway” mode of action (6–8). Herbal formulations, comprising numerous bioactive compounds, can simultaneously influence key processes—including inflammation, metabolism, and fibrosis—making them theoretically well-suited for intervening in the complex network of CMS (8). However, this very complexity presents a major scientific challenge: the mechanisms of action are difficult to elucidate, optimal patient populations are hard to define, and traditional empirical knowledge requires translation into rigorous, verifiable evidence (7–9). Addressing these gaps is essential for integrating phytotherapy into modern precision medicine.

### 1.3 Core argument and article roadmap

This perspective article posits that multi-omics biomarkers are pivotal for deciphering the systemic effects of herbal medicine and enabling its precise application in CMS-related remodeling. Multi-omics approaches provide a comprehensive framework to map biological responses at genomic, transcriptomic, proteomic, metabolomic, and microbiomic levels (10, 11).

**Figure 1:**
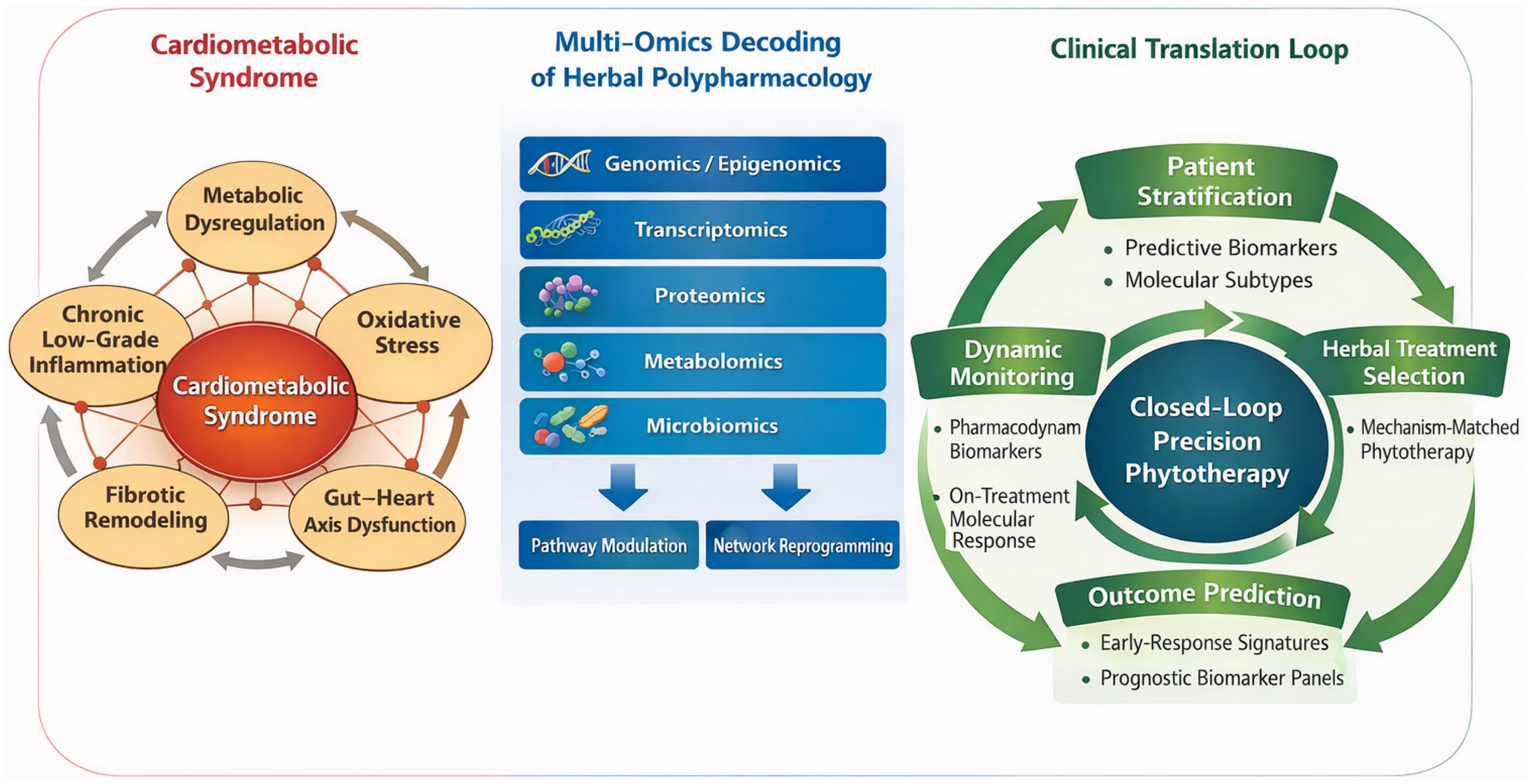
A biomarker-driven framework for precision phytotherapy in cardiometabolic syndrome.

The perspective article will first examine how multi-omics strategies can clarify the holistic mechanisms by which herbal medicines modulate the cardiometabolic remodeling network. It will then discuss how integrated multi-omics data can identify biomarker panels for predicting therapeutic response, assessing prognosis, and distinguishing disease subtypes to guide patient stratification. Finally, a translational pathway will be outlined to show how these insights can be developed into clinically actionable tools, thereby advancing phytotherapy from an empirical practice toward an evidence-based, personalized component of precision medicine (Figure 1).

## 2 Multi-omics biomarkers: deciphering herbal medicine’s polypharmacology

### 2.1 Need for multi-omics integration

The complex pathological network of CMS cannot be adequately captured by traditional single-biomarker approaches, which measure isolated analytes such as specific cytokines or lipid fractions (12, 13). These conventional markers reflect only a limited snapshot of the disease process and are incapable of representing the network-wide, synergistic effects inherent to multi-target herbal interventions (13, 14). A systems-level understanding requires the integration of multi-omics data.

This integrated approach connects biological information across complementary layers: genomics and epigenomics provide insight into genetic susceptibility and inter-individual variability (11, 15); transcriptomics and proteomics reveal dynamic changes in signaling pathways and cellular function; metabolomics captures the downstream phenotypic consequences in energy and lipid metabolism (10); and microbiomics, particularly of the gut microbiota, elucidates the role of the gut-heart axis in modulating systemic inflammation and metabolism (16). Together, these layers construct a multidimensional biological network map, enabling a holistic view of how herbal medicines restore systemic homeostasis (15).

### 2.2 Uncovering herbal mechanisms with multi-omics networks

Multi-omics technologies are uniquely suited to delineate the polypharmacological mechanisms of herbal medicines (17). This approach is particularly essential in phytotherapy, as it moves beyond the single-nutrient focus of precision nutrition by analyzing the phytochemical matrices characteristic of herbal interventions (18). These complex matrices, composed of multiple diverse constituents, exert biological effects through synergistic or additive interactions that cannot be fully attributed to individual compounds (19). Consequently, multi-omics methodologies are required to enable a system-wide analysis of the coordinated molecular responses these matrices elicit across interconnected pathways (20).

Importantly, the therapeutic relevance of such findings often lies not in pinpointing one dominant molecular target, but in uncovering co-regulated molecular modules shaped by the integrated actions of phytochemical matrices (21). This coordinated, network-level modulation is frequently missed in reductionist experimental designs, which underscores why multi-omics integration is not just beneficial but essential for a mechanistic understanding of herbal interventions (22).

This integrative paradigm is illustrated across various physiological domains. For instance, metabolomic studies of berberine (from *Coptis chinensis*) demonstrate its ability to normalize profiles associated with insulin resistance and hepatic steatosis, while transcriptomic and proteomic analyses link these effects to the modulation of AMPK and PPAR signaling pathways—illustrating a coordinated metabolic reprogramming (23, 24). Inflammation-related pathways are similarly clarified through multi-omics. Proteomic and cytokine profiling of tanshinones (from *Salvia miltiorrhiza*) show concurrent suppression of NF-κB and NLRP3 inflammasome activity, supporting a multi-target anti-inflammatory mechanism (25, 26). For fibrotic remodeling, astragaloside IV (from *Astragalus*) has been shown via integrated transcriptomic and epigenomic analyses to downregulate TGF-*β*/Smad-driven gene expression while altering histone modifications, indicating both direct and epigenetic anti-fibrotic actions (27, 28).

Moreover, many herbs exert systemic effects via the gut-heart axis. Multi-omics studies combining microbiomics and metabolomics reveal that certain herbal treatments can enrich butyrate-producing bacteria, leading to increased circulating short-chain fatty acids that subsequently attenuate inflammation and improve endothelial function (29–31). These examples demonstrate how multi-omics mapping uncovers the pleiotropic, system-level activities of herbal medicine.

### 2.3 Biomarker signatures: bridging discovery and clinical use

The translation of multi-omics insights into clinical tools hinges on the identification of validated biomarker signatures—composite indicators that reflect specific biological functions (32) (Table 1). In phytotherapy research, these signatures should be specifically viewed as functional outputs of multi-component botanical extracts, rather than as indicators of isolated compound activity (33). Integrated signatures-combining metabolites, regulatory RNAs, and signaling proteins-are especially valuable for capturing network-level biological effects (34). This approach establishes a biologically meaningful link between complex herbal formulations and their clinical outcomes (35). Accordingly, two primary and complementary types of biomarker signatures are essential for advancing precision phytotherapy.

**Table 1:** Types of multi-omics biomarker signatures and their roles in precision phytotherapy for CMS.

Biomarker signature type	Multi-omics components	Clinical purpose	Example application
Pharmacodynamic signature	Proteins, miRNAs, metabolites	Assess target engagement	Anti-inflammatory response monitoring
Predictive stratification signature	Genomics, proteomics, microbiome	Patient subtyping	Inflammation-dominant CMS
Dynamic monitoring markers	Metabolites, circulating RNAs	Dose optimization	Early on-treatment adjustment
Prognostic/early-response signature	Fibrosis-related miRNAs, metabolites	Outcome prediction	LV remodeling risk

CMS, cardiometabolic syndrome; RNA, ribonucleic acid; miRNA, microRNA; LV, left ventricular.

First, pharmacodynamic biomarker signatures quantify the modulation of key pathological pathways. For an herb targeting inflammation-dominant CMS, a signature might consist of a panel of phosphorylated signaling proteins, selected microRNAs, and inflammation-related metabolites (36, 37). Monitoring the dynamic changes in such an integrated signature provides an early and objective assessment of both target engagement and the ensuing network-level biological response, directly reflecting the extract’s polypharmacology.

Second, predictive stratification signatures identify patient subtypes most likely to respond to specific herbal interventions (38). By integrating baseline multi-omics features—such as genetic variants, proteomic profiles, and gut microbial enterotypes—patients can be classified into etiological subtypes (e.g., “metabolic-dysregulation,” “chronic-inflammation,” or “gut-dysbiosis” predominant) (39, 40). These subtypes can then guide the selection of herbs with complementary mechanisms, enabling a stratified, precision approach that moves beyond empirical prescription toward biomarker-informed herbal therapy (38, 39).

## 3 Toward precision phytotherapy: building a biomarker-driven clinical pathway

### 3.1 Patient stratification and personalized treatment protocols

The precise application of phytotherapy begins with stratifying CMS patients using objective biomarkers. Integrated multi-omics signatures—such as molecular profiles reflecting baseline metabolic dysregulation, chronic inflammation, or gut-heart axis dysfunction—can classify patients into molecular subtypes (e.g., “metabolism-dominant” or “inflammation-dominant”) (41, 42). This biological stratification can be combined with traditional Chinese medicine (TCM) syndrome patterns to create a dual diagnostic framework that enhance personalization (43). This biomarker-guided alignment between phytochemical matrices and disease subtypes distinguishes precision phytotherapy from precision nutrition paradigms, which typically optimize nutrient intake based on metabolic efficiency or deficiency states (44). In contrast, precision phytotherapy aims at pathophysiological network remodeling, leveraging the inherent polypharmacology of multi-herb matrices to restore system-level homeostasis (7, 17). Within this refined paradigm, an integrated diagnostic profile directly informs the selection of a targeted herbal formula (45). For instance, a patient with an “inflammation-dominant” biomarker profile and a TCM pattern of “phlegm-stasis intermingling” may respond optimally to a formula rich in anti-inflammatory, circulation-promoting components such as tanshinones (46). This integrated approach provides a scientific basis for translating TCM’s “syndrome differentiation” into a measurable, reproducible precision-medicine model.

### 3.2 Dynamic monitoring and dose optimization

Precision in herbal therapy requires ongoing adjustment based on treatment response. Serially measurable multi-omics biomarkers—including circulating metabolites, microRNA panels, or inflammatory proteins—enable “on-treatment monitoring” (47). These dynamic markers provide an early, objective indication of how well an herbal regimen modulates key pathological pathways, such as insulin signaling or inflammatory networks (48). By tracking changes over time, clinicians can assess whether the current dose achieves the intended biological effect, allowing for timely, evidence-based adjustments to dosage, formulation, or duration (48). This closed-loop approach moves beyond reliance on late clinical endpoints toward real-time, biomarker-guided optimization (49).

### 3.3 Predicting response and evaluating prognosis

Long-term clinical outcomes—such as reduced cardiac remodeling or fewer cardiovascular events—are the ultimate goals but take time to assess. Early changes in multi-omics biomarkers (e.g., within weeks of treatment) can serve as “early-response signatures” that predict long-term benefit (50, 51). For instance, a rapid decrease in fibrosis-related microRNAs or collagen metabolites may forecast subsequent improvements in left ventricular mass (52). Using these early signatures to identify likely non-responders allows for timely intervention, while also offering surrogate endpoints that could accelerate the clinical development of herbal therapies (52).

### 3.4 Challenges and the need for standardization

Translating this biomarker-driven vision into practice faces several hurdles. First, herbal product quality—ensuring chemical consistency and standardization—is essential for reproducible research and reliable clinical effects (53). Second, integrating and interpreting large, heterogeneous multi-omics datasets require robust bioinformatic tools and standardized analytical workflows (34). Third, the predictive value of biomarker signatures and their impact on hard clinical outcomes should be validated in large, prospective trials (54). Finally, regulatory and reimbursement frameworks need to adapt to accommodate multi-target herbal interventions and companion diagnostics, ensuring that biomarker-guided phytotherapy can be sustainably implemented in clinical care (55, 56).

## 4 Future perspectives: a roadmap for next-generation precision phytotherapy

### 4.1 Core research paradigm: closing the discovery-to-validation loop

Future research should establish a continuous, hypothesis-driven cycle that integrates discovery with validation. Beginning with evidence-informed hypotheses—often rooted in traditional knowledge—researchers can employ multi-omics technologies to uncover mechanisms and identify candidate biomarkers (57, 58). These biomarkers should then be prospectively validated in rigorously designed clinical trials where patient allocation is guided by baseline multi-omics profiles (58). The results of such trials will refine the original hypotheses, creating an iterative, self-correcting scientific framework (59). Key to this approach is the adoption of biomarker-stratified trial designs, such as basket or umbrella trials, in which patients are assigned to specific herbal interventions based on pre-defined molecular signatures (60, 61). This design directly tests whether biomarkers can predict therapeutic response, moving precision phytotherapy from concept to practice.

### 4.2 Technology integration and infrastructure development

Realizing this vision depends on the strategic integration of advanced computational and monitoring technologies. Machine-learning methods, including graph neural networks, are essential for modeling complex, nonlinear relationships within high-dimensional multi-omics datasets—going beyond conventional statistical approaches to reveal higher-order biomarker patterns (62). In parallel, wearable sensors that track continuous physiological signals (e.g., glucose variability, vascular tone) can be combined with periodic multi-omics profiling to generate dynamic, individualized “digital twin” models (63). These models would allow real-time, data-driven treatment adjustments. Equally important is the establishment of open, standardized biorepositories that link herbal product characterization, longitudinal multi-omics data, and clinical outcomes (34). Such shared resources will accelerate discovery, foster reproducibility, and support global collaboration.

### 4.3 Building an interdisciplinary ecosystem

Successful translation requires a cohesive, cross-disciplinary ecosystem. A new field of “systems-precision phytomedicine” should be cultivated, uniting experts in phytochemistry, bioinformatics, clinical trial design, and traditional medicine from the earliest stages of research planning (64). In parallel, regulatory frameworks should evolve to support biomarker-driven development pathways (65). Adaptive licensing models—which grant conditional approval for herbal products that demonstrate efficacy in biomarker-defined subpopulations, contingent on continued post-marketing evidence generation—could provide a viable route to market while ensuring scientific rigor and patient safety (66). Together, these efforts will create an enabling environment for the responsible and effective integration of precision phytotherapy into modern healthcare.

## 5 Summary

### 5.1 Summary of core arguments

Cardiovascular remodeling in cardiometabolic syndrome is systemic and heterogeneous, requiring precision interventions that target the underlying network pathology. Herbal medicine, with its inherent multi-target activity, offers a promising resource. However, fully translating its potential demands rigorous scientific translation, where multi-omics biomarkers are central. These biomarkers enable the quantification and systemic mapping of herbal effects across molecular networks, providing an evidence-based path toward standardized, reproducible phytotherapy.

### 5.2 Significance of the paradigm shift

This work outlines a transition from empirical, disease-centered herbal use to biomarker-defined precision phytotherapy. By linking multi-omics-derived molecular phenotypes with tailored herbal regimens, the approach moves beyond a “one-size-fits-all” model. This shift not only aims to improve efficacy and safety but also integrates traditional herbal wisdom with the measurable, predictive, and personalized framework of modern precision medicine.

### 5.3 Final outlook

Implementing this framework could redefine cardiovascular disease management—bridging systems-level mechanism, dynamic biomarker monitoring, and individualized treatment. The resulting strategy would transcend the limits of both single-target drugs and unstratified herbal therapy. Ultimately, this synthesis of traditional knowledge and contemporary science may offer a actionable blueprint for “precision remodeling” in cardiovascular health, uniting Eastern and Western medical insights in a forward-looking model of care.

## Data Availability

The original contributions presented in the study are included in the article/supplementary material, further inquiries can be directed to the corresponding author/s.

## References

[1] Sanz RL Inserra F García Menéndez S Mazzei L Ferder L Manucha W. Metabolic syndrome and cardiac remodeling due to mitochondrial oxidative stress involving Gliflozins and Sirtuins. Curr Hypertens Rep. (2023) 25:91–106. doi: 10.1007/s11906-023-01240-w, 37052810

[2] Khan AR Salama AH Aleem Z Alfakeer H Alnemr L Shareef AMM. The promising frontier of cardiometabolic syndrome: a new paradigm in cardiology. Cureus. (2023) 15:e45542. doi: 10.7759/cureus.45542, 37868505 PMC10586230

[3] Silveira Rossi JL Barbalho SM Reverete de Araujo R Bechara MD Sloan KP Sloan LA. Metabolic syndrome and cardiovascular diseases: going beyond traditional risk factors. Diabetes Metab Res Rev. (2022) 38:e3502. doi: 10.1002/dmrr.3502, 34614543

[4] Suffee N Le Goff W Chen J. Editorial: Cardiometabolic diseases and inflammatory responses. Front Immunol. (2024) 15:1384022. doi: 10.3389/fimmu.2024.1384022, 38495875 PMC10940500

[5] Ashfield S Ojha U. Cardiometabolic dysregulation and heart failure. Rev Cardiovasc Med. (2025) 26:38504. doi: 10.31083/RCM38504, 40475725 PMC12135638

[6] Liu Y Wang MW. Botanical drugs: challenges and opportunities: contribution to Linnaeus memorial symposium 2007. Life Sci. (2008) 82:445–9. doi: 10.1016/j.lfs.2007.11.007, 18177674

[7] Lee M Shin H Park M Kim A Cha S Lee H. Systems pharmacology approaches in herbal medicine research: a brief review. BMB Rep. (2022) 55:417–28. doi: 10.5483/BMBRep.2022.55.9.102, 35880436 PMC9537023

[8] Yang HY Liu ML Luo P Yao XS Zhou H. Network pharmacology provides a systematic approach to understanding the treatment of ischemic heart diseases with traditional Chinese medicine. Phytomedicine. (2022) 104:154268. doi: 10.1016/j.phymed.2022.154268, 35777118

[9] Shaito A Thuan DTB Phu HT Nguyen THD Hasan H Halabi S . Herbal medicine for cardiovascular diseases: efficacy, mechanisms, and safety. Front Pharmacol. (2020) 11:422. doi: 10.3389/fphar.2020.00422, 32317975 PMC7155419

[10] Leon-Mimila P Wang J Huertas-Vazquez A. Relevance of multi-omics studies in cardiovascular diseases. Front Cardiovasc Med. (2019) 6:91. doi: 10.3389/fcvm.2019.00091, 31380393 PMC6656333

[11] Babu M Snyder M. Multi-omics profiling for health. Mol Cell Proteomics. (2023) 22:100561. doi: 10.1016/j.mcpro.2023.100561, 37119971 PMC10220275

[12] Scola L Giarratana RM Torre S Argano V Lio D Balistreri CR. On the road to accurate biomarkers for cardiometabolic diseases by integrating precision and gender medicine approaches. Int J Mol Sci. (2019) 20:6015. doi: 10.3390/ijms20236015, 31795333 PMC6929083

[13] Wang RS Maron BA Loscalzo J. Multiomics network medicine approaches to precision medicine and therapeutics in cardiovascular diseases. Arterioscler Thromb Vasc Biol. (2023) 43:493–503. doi: 10.1161/ATVBAHA.122.318731, 36794589 PMC10038904

[14] Joshi A Rienks M Theofilatos K Mayr M. Systems biology in cardiovascular disease: a multiomics approach. Nat Rev Cardiol. (2021) 18:313–30. doi: 10.1038/s41569-020-00477-1 33340009

[15] Chen C Wang J Pan D Wang X Xu Y Yan J . Applications of multi-omics analysis in human diseases. MedComm. (2020). 2023) 4:e315. doi: 10.1002/mco2.315 PMC10390758 37533767

[16] Doran S Arif M Lam S Bayraktar A Turkez H Uhlen M . Multi-omics approaches for revealing the complexity of cardiovascular disease. Brief Bioinform. (2021) 22:bbab061. doi: 10.1093/bib/bbab061, 33725119 PMC8425417

[17] Zhang GB Li QY Chen QL Su SB. Network pharmacology: a new approach for chinese herbal medicine research. Evid Based Complement Alternat Med. (2013) 2013:621423. doi: 10.1155/2013/621423, 23762149 PMC3671675

[18] Zhou X Seto SW Chang D Kiat H Razmovski-Naumovski V Chan K . Synergistic effects of Chinese herbal medicine: a comprehensive review of methodology and current research. Front Pharmacol. (2016) 7:201. doi: 10.3389/fphar.2016.00201 27462269 PMC4940614

[19] Wang X Xu X Tao W Li Y Wang Y Yang L. A systems biology approach to uncovering pharmacological synergy in herbal medicines with applications to cardiovascular disease. Evid Based Complement Alternat Med. (2012) 2012:519031. doi: 10.1155/2012/519031, 23243453 PMC3518963

[20] Zhao P Li J Yang L Li Y Tian Y Li S. Integration of transcriptomics, proteomics, metabolomics and systems pharmacology data to reveal the therapeutic mechanism underlying Chinese herbal Bufei Yishen formula for the treatment of chronic obstructive pulmonary disease. Mol Med Rep. (2018) 17:5247–57. doi: 10.3892/mmr.2018.8480, 29393428 PMC5865990

[21] Zhang P Zhang D Zhou W Wang L Wang B Zhang T . Network pharmacology: towards the artificial intelligence-based precision traditional Chinese medicine. Brief Bioinform. (2023) 25:bbad518. doi: 10.1093/bib/bbad518, 38197310 PMC10777171

[22] Lv S Wang Q Zhang X Ning F Liu W Cui M . Mechanisms of multi-omics and network pharmacology to explain traditional chinese medicine for vascular cognitive impairment: a narrative review. Phytomedicine. (2024) 123:155231. doi: 10.1016/j.phymed.2023.155231, 38007992

[23] Li J Liu Z Guo M Xu K Jiang M Lu A . Metabolomics profiling to investigate the pharmacologic mechanisms of berberine for the treatment of high-fat diet-induced nonalcoholic steatohepatitis. Evid Based Complement Alternat Med. (2015) 2015:897914. doi: 10.1155/2015/897914, 25977701 PMC4421035

[24] Zhu X Bian H Wang L Sun X Xu X Yan H . Berberine attenuates nonalcoholic hepatic steatosis through the AMPK-SREBP-1c-SCD1 pathway. Free Radic Biol Med. (2019) 141:192–204. doi: 10.1016/j.freeradbiomed.2019.06.019, 31226399

[25] Zhao J Liu H Hong Z Luo W Mu W Hou X . Tanshinone I specifically suppresses NLRP3 inflammasome activation by disrupting the association of NLRP3 and ASC. Mol Med. (2023) 29:84. doi: 10.1186/s10020-023-00671-0, 37400760 PMC10318668

[26] Chen Z Gao X Jiao Y Qiu Y Wang A Yu M . Tanshinone IIA exerts anti-inflammatory and immune-regulating effects on vulnerable atherosclerotic plaque partially via the TLR4/MyD88/NF-κB signal pathway. Front Pharmacol. (2019) 10:850. doi: 10.3389/fphar.2019.00850, 31402870 PMC6677033

[27] Shi L Deng J He J Zhu F Jin Y Zhang X . Integrative transcriptomics and proteomics analysis reveal the protection of Astragaloside IV against myocardial fibrosis by regulating senescence. Eur J Pharmacol. (2024) 975:176632. doi: 10.1016/j.ejphar.2024.176632, 38718959

[28] Qian W Cai X Qian Q Zhang W Wang D. Astragaloside IV modulates TGF-β1-dependent epithelial-mesenchymal transition in bleomycin-induced pulmonary fibrosis. J Cell Mol Med. (2018) 22:4354–65. doi: 10.1111/jcmm.13725, 29971947 PMC6111865

[29] Zhang X Zhao Y Xu J Xue Z Zhang M Pang X . Modulation of gut microbiota by berberine and metformin during the treatment of high-fat diet-induced obesity in rats. Sci Rep. (2015) 5:14405. doi: 10.1038/srep14405, 26396057 PMC4585776

[30] Ohira H Tsutsui W Fujioka Y. Are short chain fatty acids in gut microbiota defensive players for inflammation and atherosclerosis? J Atheroscler Thromb. (2017) 24:660–72. doi: 10.5551/jat.RV17006, 28552897 PMC5517538

[31] Tian Q Leung FP Chen FM Tian XY Chen Z Tse G . Butyrate protects endothelial function through PPARδ/miR-181b signaling. Pharmacol Res. (2021) 169:105681. doi: 10.1016/j.phrs.2021.105681, 34019979

[32] Califf RM. Biomarker definitions and their applications. Exp Biol Med (Maywood). (2018) 243:213–21. doi: 10.1177/1535370217750088, 29405771 PMC5813875

[33] Zhao M Che Y Gao Y Zhang X. Application of multi-omics in the study of traditional Chinese medicine. Front Pharmacol. (2024) 15:1431862. doi: 10.3389/fphar.2024.1431862, 39309011 PMC11412821

[34] Xie C Che Y Zhou Y Wu J Shen J. Multi-omics research strategies in traditional Chinese medicine: a review. Medicine. (2025) 104:e45479. doi: 10.1097/MD.0000000000045479, 41261578 PMC12582753

[35] Wang D Li R Wei S Gao S Xu Z Liu H . Metabolomics combined with network pharmacology exploration reveals the modulatory properties of Astragali Radix extract in the treatment of liver fibrosis. Chin Med. (2019) 14:30. doi: 10.1186/s13020-019-0251-z, 31467589 PMC6712842

[36] Bachstetter AD Watterson DM Van Eldik LJ. Target engagement analysis and link to pharmacodynamic endpoint for a novel class of CNS-penetrant and efficacious p38α MAPK inhibitors. J Neuroimmune Pharmacol. (2014) 9:454–60. doi: 10.1007/s11481-014-9543-3, 24789302 PMC4122817

[37] Batra SK Heier CR Diaz-Calderon L Tully CB Fiorillo AA van den Anker J . Serum miRNAs are pharmacodynamic biomarkers associated with therapeutic response in pediatric inflammatory bowel disease. Inflamm Bowel Dis. (2020) 26:1597–606. doi: 10.1093/ibd/izaa209, 32793975 PMC7500519

[38] Hasin Y Seldin M Lusis A. Multi-omics approaches to disease. Genome Biol. (2017) 18:83. doi: 10.1186/s13059-017-1215-1, 28476144 PMC5418815

[39] Karczewski KJ Snyder MP. Integrative omics for health and disease. Nat Rev Genet. (2018) 19:299–310. doi: 10.1038/nrg.2018.4, 29479082 PMC5990367

[40] Costea PI Hildebrand F Arumugam M Bäckhed F Blaser MJ Bushman FD . Enterotypes in the landscape of gut microbial community composition. Nat Microbiol. (2018) 3:8–16. doi: 10.1038/s41564-017-0072-8, 29255284 PMC5832044

[41] Chen D Zhao X Sui Z Niu H Chen L Hu C . A multi-omics investigation of the molecular characteristics and classification of six metabolic syndrome relevant diseases. Theranostics. (2020) 10:2029–46. doi: 10.7150/thno.41106, 32089734 PMC7019171

[42] Bianchi J Duarte FO Camillo L Godoy KF Rodolpho JMA Fragelli BDL . Cluster analysis reveals distinct inflammatory phenotypes in cardiometabolic disease. Cardiovasc Diabetol Endocrinol Rep. (2025) 11:15. doi: 10.1186/s40842-025-00227-7, 41013857 PMC12320283

[43] Wu G Zhao J Zhao J Song N Zheng N Zeng Y . Exploring biological basis of syndrome differentiation in coronary heart disease patients with two distinct syndromes by integrated multi-omics and network pharmacology strategy. Chin Med. (2021) 16:109. doi: 10.1186/s13020-021-00521-3, 34702323 PMC8549214

[44] de Toro-Martín J Arsenault BJ Després JP Vohl MC. Precision nutrition: a review of personalized nutritional approaches for the prevention and Management of Metabolic Syndrome. Nutrients. (2017) 9:913. doi: 10.3390/nu9080913, 28829397 PMC5579706

[45] Li J Lu C Jiang M Niu X Guo H Li L . Traditional chinese medicine-based network pharmacology could lead to new multicompound drug discovery. Evid Based Complement Alternat Med. (2012) 2012:149762. doi: 10.1155/2012/149762, 23346189 PMC3541710

[46] Shang H Zhang L Xiao T Zhang L Ruan J Zhang Q . Study on the differences of gut microbiota composition between phlegm-dampness syndrome and qi-yin deficiency syndrome in patients with metabolic syndrome. Front Endocrinol (Lausanne). (2022) 13:1063579. doi: 10.3389/fendo.2022.1063579, 36440222 PMC9682026

[47] Stolaki EV Psatha K Aivaliotis M. Metabolomics and pharmacometabolomics: advancing precision medicine in drug discovery and development. Meta. (2025) 15:750. doi: 10.3390/metabo15110750, 41295335 PMC12654014

[48] Schmidt JC Dougherty BV Beger RD Jones DP Schmidt MA Mattes WB. Metabolomics as a truly translational tool for precision medicine. Int J Toxicol. (2021) 40:413–26. doi: 10.1177/10915818211039436, 34514887 PMC8443142

[49] Rankin NJ Preiss D Welsh P Sattar N. Applying metabolomics to cardiometabolic intervention studies and trials: past experiences and a roadmap for the future. Int J Epidemiol. (2016) 45:1351–71. doi: 10.1093/ije/dyw271, 27789671 PMC5100629

[50] Gao G Chen W Liu M Yan X Yang P. Circulating MicroRNAs as novel potential biomarkers for left ventricular remodeling in postinfarction heart failure. Dis Markers. (2019) 2019:5093803. doi: 10.1155/2019/5093803, 31885737 PMC6914954

[51] Iacobescu L Ciobanu AO Macarie R Vadana M Ciortan L Tucureanu MM . Diagnostic and prognostic role of circulating microRNAs in patients with coronary artery disease-impact on left ventricle and arterial function. Curr Issues Mol Biol. (2024) 46:8499–511. doi: 10.3390/cimb46080500, 39194717 PMC11352712

[52] Alzaabi MA Abdelsalam A Alhammadi M Bani Hani H Almheiri A Al Matrooshi N . Evaluating biomarkers as tools for early detection and prognosis of heart failure: a comprehensive review. Card Fail Rev. (2024) 10:e06. doi: 10.15420/cfr.2023.24, 38915376 PMC11194781

[53] Zhang J Wider B Shang H Li X Ernst E. Quality of herbal medicines: challenges and solutions. Complement Ther Med. (2012) 20:100–6. doi: 10.1016/j.ctim.2011.09.004 22305255

[54] Li Y Xu Z Du P Gao J Wang S Pang X . Methodological challenges in pilot trials of herbal medicine: barriers to evidence-based practice. J Clin Epidemiol. (2025) 182:111754. doi: 10.1016/j.jclinepi.2025.111754, 40081675

[55] Parveen A Parveen B Parveen R Ahmad S. Challenges and guidelines for clinical trial of herbal drugs. J Pharm Bioallied Sci. (2015) 7:329–33. doi: 10.4103/0975-7406.168035, 26681895 PMC4678978

[56] Dubale S Usure RE Mekasha YT Hasen G Hafiz F Kebebe D . Traditional herbal medicine legislative and regulatory framework: a cross-sectional quantitative study and archival review perspectives. Front Pharmacol. (2025) 16:1475297. doi: 10.3389/fphar.2025.1475297, 39950109 PMC11821589

[57] Park JJH Hsu G Siden EG Thorlund K Mills EJ. An overview of precision oncology basket and umbrella trials for clinicians. CA Cancer J Clin. (2020) 70:125–37. doi: 10.3322/caac.21600, 32031692 PMC7187272

[58] Duan XP Qin BD Jiao XD Liu K Wang Z Zang YS. New clinical trial design in precision medicine: discovery, development and direction. Signal Transduct Target Ther. (2024) 9:57. doi: 10.1038/s41392-024-01760-0, 38438349 PMC10912713

[59] Jiang Z Zhang H Gao Y Sun Y. Multi-omics strategies for biomarker discovery and application in personalized oncology. Mol Biomed. (2025) 6:115. doi: 10.1186/s43556-025-00340-0, 41269529 PMC12638490

[60] Superchi C Brion Bouvier F Gerardi C Carmona M San Miguel L Sánchez-Gómez LM . Study designs for clinical trials applied to personalised medicine: a scoping review. BMJ Open. (2022) 12:e052926. doi: 10.1136/bmjopen-2021-052926, 35523482 PMC9083424

[61] Fountzilas E Tsimberidou AM Vo HH Kurzrock R. Clinical trial design in the era of precision medicine. Genome Med. (2022) 14:101. doi: 10.1186/s13073-022-01102-1, 36045401 PMC9428375

[62] Lin M Guo J Gu Z Tang W Tao H You S . Machine learning and multi-omics integration: advancing cardiovascular translational research and clinical practice. J Transl Med. (2025) 23:388. doi: 10.1186/s12967-025-06425-2, 40176068 PMC11966820

[63] Ooka T. The era of preemptive medicine: developing medical digital twins through omics, IoT, and AI integration. JMA J. (2025) 8:1–10. doi: 10.31662/jmaj.2024-0213, 39926086 PMC11799569

[64] Thomford NE Dzobo K Chimusa E Andrae-Marobela K Chirikure S Wonkam A . Personalized herbal medicine? A roadmap for convergence of herbal and precision medicine biomarker innovations. OMICS. (2018) 22:375–91. doi: 10.1089/omi.2018.0074, 29927715

[65] Hua H Tang JY Zhao JN Wang T Zhang JH Yu JY . From traditional medicine to modern medicine: the importance of TCM regulatory science (TCMRS) as an emerging discipline. Chin Med. (2025) 20:92. doi: 10.1186/s13020-025-01152-8, 40563101 PMC12199503

[66] Knowles L Luth W Bubela T. Paving the road to personalized medicine: recommendations on regulatory, intellectual property and reimbursement challenges. J Law Biosci. (2017) 4:453–506. doi: 10.1093/jlb/lsx030, 29868182 PMC5965495

